# Interpretable machine learning for severity classification of thyroid eye disease using orbital anatomical features

**DOI:** 10.3389/fmed.2026.1840814

**Published:** 2026-06-19

**Authors:** Ruixin Shi, Leiming Gao, Shengzhi Jiao, Liuzi Wang, Jianing Li, Bei Wang

**Affiliations:** 1 School of Nursing, Nanjing University of Chinese Medicine, Nanjing, Jiangsu, China; 2 Department of Nursing, Affiliated Hospital of Traditional Chinese and Western Medicine, Nanjing University of Chinese Medicine, Nanjing, Jiangsu, China

**Keywords:** thyroid eye disease, disease severity, feature importance, machine learning, magnetic resonance imaging, orbital anatomy

## Abstract

**Background:**

Thyroid eye disease (TED) severity assessment using the EUGOGO severity classification is partly subjective and prone to interobserver variability. While MRI-derived anatomical measurements offer objective features, such as ocular protrusion and extraocular muscle thickness, these are underutilized in machine learning (ML) models that often rely on non-interpretable radiomic features. Moreover, the inclusion of longitudinal scans from the same patient may artificially inflate model performance due to unaccounted intra-patient correlations and temporal redundancy.

**Purpose:**

To develop an interpretable machine learning framework for objective TED severity stratification (mild, moderate-to-severe, sight-threatening) by quantitatively integrating established orbital anatomical parameters aligned with clinical assessment criteria, and to evaluate how data handling strategies influence model generalizability.

**Methods:**

We retrospectively analyzed 443 patients with TED, yielding 1,054 orbital MRI units from all available examinations. Two datasets were constructed: Dataset A included all available scans, while Dataset B retained only the first-visit orbital MRI units per patient (886 orbital units) to reduce temporal and repeat-visit bias. As bilateral orbits from the same patient were included, Dataset B reduces visit-related confounding but retains inherent inter-eye correlations. Six ML models—Logistic Regression (LR), Support Vector Machine (SVM), K-Nearest Neighbors (KNN), Random Forest (RF), XGBoost, and LightGBM—were trained and evaluated using cross-validation. Model performance was compared in terms of area under the receiver operating characteristic curve (AUC), F1-score, and recall. Feature importance was assessed using RF, XGBoost, and LightGBM.

**Results:**

Under class-imbalance strategies, Random Forest with class weighting achieved the highest AUC (0.811). Random Forest with SMOTE achieved the highest recall (0.669), F1-score (0.648), and specificity (0.815). Performance on Dataset A demonstrated how unaccounted longitudinal scan correlations can inflate metrics, reinforcing the necessity of temporal deduplication and cautious handling of orbital-level correlations. Feature importance analysis consistently ranked ocular protrusion as the top predictor, followed by rectus muscle thicknesses and orbital geometric parameters.

**Conclusion:**

Controlling for longitudinal redundancy and intra-patient correlations significantly impacts model evaluation and generalizability. Random Forest with class weighting demonstrated the best discriminative performance in our internal validation on temporally deduplicated first-visit scans. Rather than relying on isolated diagnostic thresholds, the framework integrates measurable anatomical parameters to generate predictions that complement categorical clinical grading. This approach emphasizes standardized quantification and workflow reproducibility, highlighting the need for rigorous data structuring and transparent feature alignment in medical AI.

## Introduction

Thyroid eye disease (TED), also known as Graves’ ophthalmopathy, is one of the most common orbital diseases in adults and represents the primary ocular manifestation of autoimmune thyroid disease (1). It is characterized pathologically by inflammatory hyperplasia of the extraocular muscles and intra-orbital adipose tissue, leading to a spectrum of clinical manifestations including proptosis, eyelid retraction, diplopia, corneal exposure, and, in severe cases, compressive optic neuropathy resulting from apical orbital crowding, potentially culminating in vision loss or even blindness (2). Beyond its impact on visual function, TED induces significant cosmetic alterations that profoundly affect patients’ psychological well-being and overall quality of life (3–7).

Currently, the clinical assessment of TED severity primarily relies on standardized clinical scoring systems, among which the European Group on Graves’ Orbitopathy (EUGOGO) severity classification is most widely used (8, 9). However, this approach is inherently subjective, being influenced by both clinician interpretation and patient-reported symptoms, leading to substantial inter-observer variability and limited reproducibility (10). Moreover, conventional clinical scoring lacks sensitivity to subtle, early anatomical changes within the orbit, thereby failing to fully capture the complex three-dimensional structural remodeling that occurs during disease progression (11). It is worth noting that the clinical manifestations of some patients are not fully consistent with the imaging changes, and this clinical and imaging mismatch further increases the complexity of the assessment (8), highlighting the need for objective and quantifiable assessment tools.

In recent years, magnetic resonance imaging (MRI) has emerged as a valuable modality in the diagnosis and monitoring of TED due to its superior soft-tissue contrast resolution (12). Studies have demonstrated that TED is associated with characteristic orbital anatomical changes (13), including symmetric or asymmetric thickening of the extraocular muscles, adipose tissue proliferation, and increased globe protrusion (14). These imaging-derived parameters are quantifiable, reproducible, and have been shown to correlate with both disease activity and severity (15). Importantly, the orbit functions as a three-dimensional biomechanical cavity composed of bony walls and soft tissues, and its global morphology may undergo dynamic remodeling throughout the course of TED (16). Beyond changes in muscle volume and fat content, precise anatomical metrics, such as the inclination angle of the bony orbital wall, the geometric relationship of the orbital rim, and the spatial angle between the common tendinous ring and critical bony landmarks, may provide additional insights into the pathophysiological mechanisms underlying disease progression. Nevertheless, most existing research has focused predominantly on muscle thickness or signal intensity on MRI (16), with limited systematic integration of these morphologically meaningful and anatomically interpretable parameters, particularly in the context of predictive modeling.

Concurrently, advances in artificial intelligence have propelled machine learning (ML) and deep learning into the forefront of ophthalmic image analysis. Recent studies have successfully applied these techniques to TED assessment, demonstrating high accuracy in binary disease detection using external photographs (17), multi-modal clinical activity scoring combining slit-lamp and facial images (18), automated phase differentiation (active vs. inactive) via MRI or CT (19, 20), and binary severity risk prediction utilizing hybrid clinical-imaging features (21). While these advances represent significant methodological progress, they predominantly address binary classification tasks or activity-based classification rather than three distinct severity tiers (mild, moderate-to-severe, sight-threatening) using reproducible MRI-derived anatomical measurements. Additionally, many recent approaches prioritize high-dimensional feature extraction to maximize predictive performance (22, 23), which may reduce direct anatomical interpretability, and retrospective dataset construction frequently incorporates longitudinal scans without explicit patient-level or visit-level partitioning (24, 25). Our study aims to complement this growing literature by focusing on transparent, reproducible orbital metrics and systematically evaluating how data handling strategies influence model reliability for three-class severity staging.

An often-overlooked aspect in real-world clinical data is the presence of longitudinal MRI scans from the same patient across multiple visits. Treating multiple follow-up scans as independent samples in machine learning modeling may violate the independence assumption underlying standard validation protocols, potentially inflating model performance, particularly for distance-based algorithms such as k-nearest neighbors (KNN) (26). Conversely, restricting analysis to only the first visit per patient reduces temporal redundancy but may limit statistical power and longitudinal insights. To date, few studies have explicitly compared how these distinct data handling strategies, incorporating all available longitudinal scans versus retaining only baseline visits, affect the robustness of TED severity prediction models in real-world clinical workflows.

The aim of this study was to integrate a series of orbital anatomical features that can be reliably measured on standard MRI planes, specifically, the mid-sagittal and axial views, including globe protrusion, thicknesses of the four major extraocular muscles (superior, inferior, medial, and lateral recti), the vertical distance from the supraorbital apex to the infraorbital wall and its angular relationship with the common tendinous ring, as well as the line connecting the bony inner and outer orbital rims and its angle relative to the optic foramen. This feature set comprises both clinically referenced metrics and supplementary quantitative parameters, intentionally selected to capture the multi-dimensional morphological changes associated with TED while maintaining measurement feasibility. Rather than relying on isolated threshold-based rules, the framework integrates these quantifiable inputs to learn non-linear feature interactions and generate objective severity predictions. This design positions the model as a standardized assessment tool that complements categorical clinical grading by reducing subjectivity in borderline cases and providing reproducible risk stratification. Combined with multiple machine learning algorithms, these measurements were used to construct and evaluate TED severity prediction models under two distinct data strategies: (1) a comprehensive dataset incorporating all available longitudinal MRI scans, and (2) a baseline-only dataset retaining only the first-visit scan per patient. This study also systematically examines how data structure, specifically the inclusion of longitudinal follow-up scans, influences model performance and generalizability. These findings offer practical insights into selecting appropriate machine learning approaches for clinical settings characterized by limited sample sizes and a strong need for model interpretability.

## Methods

### Study design and participants

This retrospective study enrolled patients diagnosed with TED from the inpatient records of the Department of Endocrinology, Thyroid and Breast Surgery, and Ophthalmology at Jiangsu Provincial Hospital of Integrated Traditional Chinese and Western Medicine, between January 2021 to December 2024.

The inclusion criteria were: (1) age ≥18 years old; (2) a clinical diagnosis of TED based on the *Chinese Guidelines for the Diagnosis and Treatment of Thyroid Eye Disease (2022)* (27) and confirmed by multidisciplinary clinician consensus; and (3) availability of complete clinical, biochemical, and orbital MRI data. Exclusion criteria included concomitant ocular inflammatory or systemic autoimmune diseases, pregnancy, or prior ocular prosthetic implantation.

All enrolled patients met at least one of the following clinical indications for orbital MRI (28, 29): (i) suspected optic nerve compression (defined as a decrease in visual acuity ≥2 Snellen lines and/or impaired color vision); (ii) Clinical Activity Score (CAS) ≥ 4; (iii) progression of proptosis ≥2 mm/month; or (iv) new-onset or worsening diplopia. Patients who underwent only CT or had incomplete MRI protocols were excluded to ensure imaging modality consistency and complete feature extraction.

To evaluate the impact of data structure on model performance, two distinct datasets were constructed. Dataset A included all eligible orbital-level observations derived from MRI examinations. Dataset B retained only the first-visit MRI-derived orbital units from each patient to reduce temporal redundancy and repeat-visit bias.

### Data collection

Clinical data and orbital MRI images were extracted from the hospital’s electronic medical record system and the Rekall Medical Image Storage and Communication System. When multiple imaging records existed, the MRI scan temporally closest to the clinical assessment was selected for analysis.

Disease severity was retrospectively classified according to the EUGOGO consensus criteria by two independent ophthalmologists blinded to the imaging metrics. Severity labels were assigned based on contemporaneous clinical findings and EUGOGO criteria, rather than on the MRI-derived anatomical measurements used as model inputs. Classification was based on clinical findings documented within the same visit cycle as the MRI examination (≤48 h) (8). Cases were excluded if the interval between clinical evaluation and MRI exceeded 48 h, or if interventions (e.g., glucocorticoids or radiotherapy) occurred during this window that could alter disease status.

MRI scans were acquired using a GE MR750 3.0 T scanner equipped with an 8-channel head coil. The protocol included the following sequences:

- Axial fat-suppressed T2-weighted imaging (T2WI): TR/TE = 4,037 ms/85 ms, slice thickness 3 mm
- Coronal fat-suppressed T2WI: TR/TE = 5,592 ms/85 ms, slice thickness 3 mm
- Axial T1-weighted imaging (T1WI): TR/TE = 486 ms/minimum full echo time, slice thickness 3 mm

A total of nine features were extracted: globe protrusion, thicknesses of the four rectus muscles, orbital height and width, and their corresponding angles (h1 and w1).

- Globe protrusion: defined as the perpendicular distance from the corneal apex to the line connecting the anterior bony margins of the orbital rim, measured on axial views.
- Extraocular muscle thickness: manually contoured on coronal T2WI at the level 1 cm posterior to the globe, selecting the maximal cross-sectional diameter of each of the four rectus muscles (superior, inferior, medial, lateral), excluding surrounding fat.
- Orbital height (h): the vertical distance from the supraorbital apex to the infraorbital wall in the mid-sagittal plane, and its angular relationship with the common tendinous ring (h1).
- Orbital width (w): the line between the bony apex of the outer orbital rim and the inner orbital margin in the mid-horizontal plane, and its angle relative to the optic foramen (w1).

### Severity classification

Disease severity was categorized using the EUGOGO classification system (8):

- Mild TED: presence of one or more of the following: eyelid retraction <2 mm, mild soft tissue involvement, proptosis <22 mm, transient or no diplopia, absence of corneal exposure, and normal optic nerve function.
- Moderate-to-Severe TED: proptosis ≥22 mm, diplopia (sustained or intermittent), moderate soft tissue signs, or corneal involvement without optic neuropathy.
- Sight-Threatening TED: evidence of dysthyroid optic neuropathy (DON), including optic nerve compression and/or severe corneal complications.

### Data preprocessing

Missing values in imaging features were imputed using the Multivariate Imputation by Chained Equations (MICE) method, which preserves data distribution and correlation structures. Categorical variables were encoded appropriately, and the training and test sets were created using stratified random sampling to preserve class proportions.

A total of nine predictive features were included: globe protrusion, thicknesses of the four rectus muscles, orbital height (h), orbital width (w), and their respective angles (h₁, w₁). The dataset was partitioned into training (80%) and test (20%) sets using stratified random sampling to maintain proportional representation of severity classes in both subsets.

### Model development

Six machine learning algorithms were implemented to predict TED severity: Logistic Regression (LR), Random Forest (RF), XGBoost, LightGBM, Support Vector Machines (SVM) and K-Nearest Neighbors (KNN).

For models that are sensitive to feature scale (LR, SVM, KNN), features normalization was performed using StandardScaler (zero mean, unit variance) based on the training set and applied consistently to the test set. Tree-based models (RF, XGBoost, LightGBM) were trained on raw features without scaling.

Given the multi-class nature of the outcome, we evaluated three strategies to address potential class imbalance during the training phase: (1) the original unweighted approach, (2) class weighting using class_weight = ‘balanced’ (or equivalent sample weighting for boosting models), and (3) over-sampling of minority classes using Synthetic Minority Over-sampling Technique (SMOTE) applied only to the training set to prevent data leakage.

Hyperparameter tuning was conducted via grid search combined with 5-fold stratified cross-validation (StratifiedKFold). The optimal model was selected based on best cross-validation performance and subsequently evaluated on the held-out test set.

For multiclass evaluation, AUC was calculated using a one-vs-rest framework and macro-averaged across classes. Precision, recall, specificity, and F1-score were also reported as macro-averaged values.

### Software and tools

All statistical analyses were performed in Python (version 3.9). Key libraries included: pandas for data manipulation; scikit-learn for machine learning workflows; XGBoost and LightGBM for gradient boosting models; and matplotlib/seaborn for visualization.

## Result

### Baseline characteristics

This study encompassed a total of 443 patients diagnosed with TED, comprising 282 females (63.66%) and 161 males (36.34%). The average age of the participants was 45.26 ± 14.12 years. Female patients were older on average than male patients. Among these patients, 54 (12.19%) had multiple visits and MRI examinations, suggesting the availability of longitudinal follow-up data.

The datasets were constructed using orbital units as the analytical basis. Dataset A included all available orbital units, totaling 1,054. Dataset B retained only the first-visit orbital units per patient to reduce repeat-visit bias, yielding 886 orbital units. The categorical distribution of TED severity across both datasets is detailed in Table 1.

**Table 1:** Categorical distribution of TED severity.

Group	Dataset A (1,054 orbital units)	Dataset B (886 orbital units)
Mild	506	423
Moderate-to-Severe	473	394
Sight-Threatening	75	69

### Feature importance analysis

**Figure 1:**
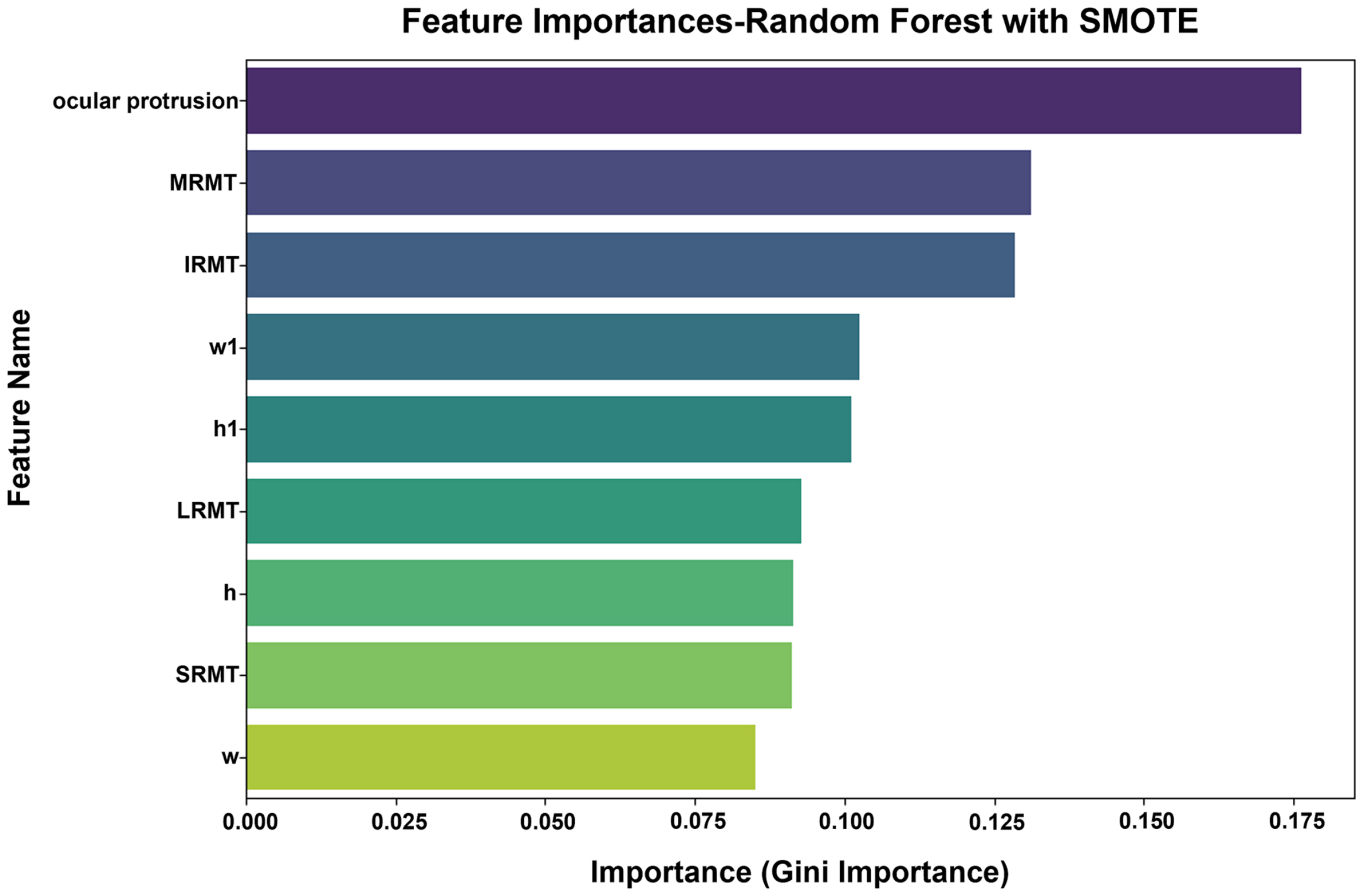
Feature importance rankings from the Random Forest model trained on Dataset A (1,054 orbital units) using the SMOTE strategy. Importance values are based on Gini impurity reduction. The top three features—ocular protrusion, MRMT, and IRMT—accounted for the majority of predictive power. MRMT, medial rectus muscle thickness; IRMT, inferior rectus muscle thickness; LRMT, lateral rectus muscle thickness; SRMT, superior rectus muscle thickness; h: Orbital height, the vertical distance from the supraorbital apex to the infraorbital wall in the mid-sagittal plane; h1, h’s angular relationship with the common tendinous ring (h1); w, Orbital width, the line between the bony apex of the outer orbital rim and the inner orbital margin in the mid-horizontal plane; w1, w’s angle relative to the optic foramen.

**Figure 2:**
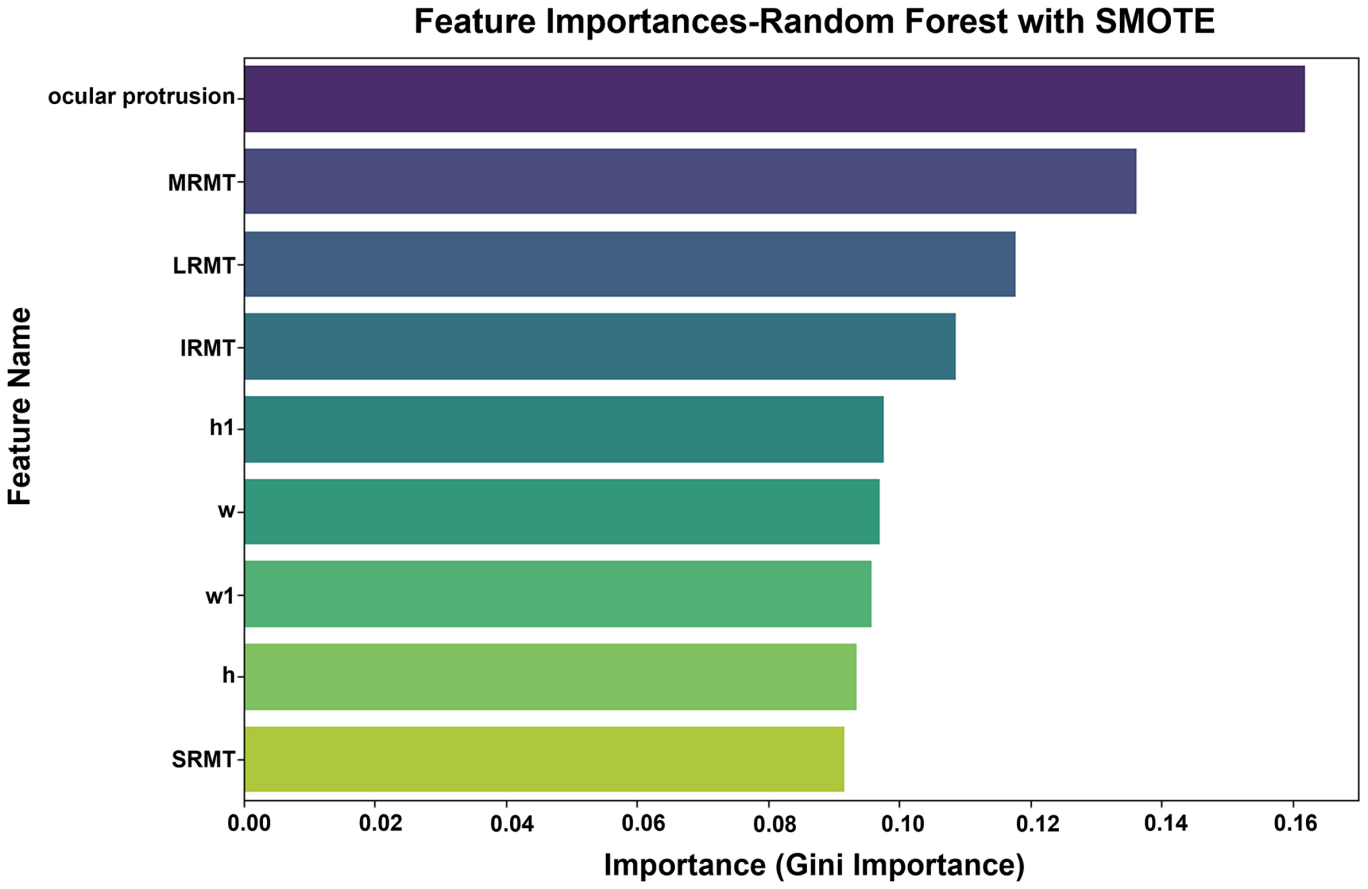
Feature importance rankings from the Random Forest model trained on Dataset B (886 orbital units) using the SMOTE strategy. Importance values are based on Gini impurity reduction. The top three features—ocular protrusion, MRMT, and IRMT—accounted for the majority of predictive power. MRMT: medial rectus muscle thickness; IRMT, inferior rectus muscle thickness; LRMT, lateral rectus muscle thickness; SRMT, superior rectus muscle thickness; h, Orbital height, the vertical distance from the supraorbital apex to the infraorbital wall in the mid-sagittal plane; h1, h’s angular relationship with the common tendinous ring (h1); w, Orbital width, the line between the bony apex of the outer orbital rim and the inner orbital margin in the mid-horizontal plane; w1, w’s angle relative to the optic foramen.

The feature importance rankings were derived from Random Forest models trained on both Dataset A (1,054 orbital units) and Dataset B (886 orbital units) under the SMOTE strategy, using Gini importance as the metric (Figures 1, 2). In both datasets, ocular protrusion was identified as the most influential feature. For Dataset A, the next highest importances were for MRMT, IRMT, followed by w1, h1, and LRMT. Features h, SRMT, and w showed relatively lower but still significant contributions.

In Dataset B, the ranking was similar with MRMT and LRMT following ocular protrusion, then IRMT, h1, and w. Features w1, h, and SRMT also contributed meaningfully, though to a slightly lesser extent. Across both datasets, all features exhibited notable importance scores, indicating their collective contribution to classification performance.

### Model performance on Dataset A

In Dataset A (1,054 orbital units), several models achieved nominally high AUC values, with KNN (original) reaching 0.807 and KNN (SMOTE) reaching 0.806. However, these may reflect temporal redundancy and inter-scan correlations inherent to longitudinal data inclusion. Under SMOTE, Random Forest achieved an F1-score of 0.576 and AUC of 0.745, while Logistic Regression exhibited the lowest performance (F1: 0.366). (Table 2).

**Table 2:** Performance comparison of six machine learning models under three class imbalance strategies using Dataset A (1,054 orbital units).

Strategy	Model	Accuracy	Precision	Recall	Specificity	F1-Score	AUC
Original	RF	0.621	0.414	0.444	0.763	0.426	0.754
	LR	0.621	0.416	0.443	0.762	0.423	0.701
	SVM	0.607	0.406	0.433	0.755	0.416	0.708
	XGBoost	0.611	0.409	0.436	0.758	0.416	0.660
	LightGBM	0.649	0.769	0.482	0.780	0.483	0.717
	KNN	0.664	0.777	0.493	0.789	0.494	0.807
Class weight	RF	0.645	0.762	0.498	0.777	0.515	0.758
	LR	0.550	0.487	0.521	0.760	0.455	0.695
	SVM	0.5833	0.470	0.473	0.759	0.467	0.690
	XGBoost	0.611	0.607	0.494	0.760	0.515	0.728
	LightGBM	0.616	0.578	0.479	0.763	0.492	0.746
SMOTE	RF	0.678	0.626	0.559	0.803	0.576	0.745
	LR	0.474	0.415	0.465	0.732	0.366	0.653
	SVM	0.616	0.534	0.554	0.778	0.541	0.727
	XGBoost	0.635	0.548	0.529	0.782	0.535	0.749
	LightGBM	0.626	0.608	0.523	0.770	0.547	0.751
	KNN	0.621	0.567	0.651	0.801	0.565	0.806

Original, no class imbalance correction applied; class weight: adjusts model loss function to assign higher penalty to misclassification of minority classes; SMOTE, synthetic minority over-sampling technique, used to balance class distribution by generating synthetic samples; AUC, the area under the receiver operating characteristic curve; RF, Random Forest; LR, logistic regression; SVM, support vector machines; KNN, K-nearest neighbors; AUC, the area under the receiver operating characteristic curve.

### Model performance on Dataset B

In Dataset B (886 orbital units, first-visit only), Random Forest consistently demonstrated robust performance across imbalance strategies. Random Forest with class weighting achieved the highest AUC (0.811). Random Forest with SMOTE yielded the highest recall (0.669), F1-score (0.648), and specificity (0.815), while the original RF achieved the highest accuracy (0.680, tied with SMOTE-RF). LightGBM under the original strategy achieved the highest precision (0.691). Logistic Regression consistently underperformed across all metrics under SMOTE (AUC: 0.714, F1: 0.436). (Table 3).

**Table 3:** Performance comparison of six machine learning models under three class imbalance strategies using Dataset B (886 orbital units).

Strategy	Model	Accuracy	Precision	Recall	Specificity	F1-Score	AUC
Original	RF	0.680	0.663	0.590	0.807	0.614	0.806
	LR	0.618	0.409	0.445	0.763	0.425	0.735
	SVM	0.629	0.417	0.453	0.770	0.432	0.754
	XGBoost	0.612	0.593	0.539	0.765	0.556	0.747
	LightGBM	0.629	0.691	0.552	0.771	0.588	0.761
	KNN	0.629	0.587	0.493	0.773	0.505	0.770
Class weight	RF	0.661	0.631	0.578	0.798	0.597	0.811
	LR	0.556	0.494	0.537	0.766	0.483	0.735
	SVM	0.579	0.513	0.553	0.775	0.502	0.733
	XGBoost	0.635	0.572	0.616	0.792	0.587	0.761
	LightGBM	0.629	0.576	0.651	0.793	0.594	0.777
SMOTE	RF	0.680	0.633	0.669	0.815	0.648	0.796
	LR	0.506	0.458	0.518	0.747	0.436	0.714
	SVM	0.590	0.539	0.605	0.770	0.558	0.748
	XGBoost	0.612	0.563	0.600	0.776	0.578	0.756
	LightGBM	0.573	0.527	0.573	0.756	0.543	0.743
	KNN	0.551	0.510	0.614	0.767	0.514	0.745

Original, no class imbalance correction applied; class weight: adjusts model loss function to assign higher penalty to misclassification of minority classes; SMOTE, synthetic minority over-sampling technique, used to balance class distribution by generating synthetic samples; AUC, the area under the receiver operating characteristic curve; RF, Random Forest; LR, logistic regression; SVM, support vector machines; KNN, K-nearest neighbors; AUC, the area under the receiver operating characteristic curve.

### Comparative analysis of model performance

**Figure 3:**
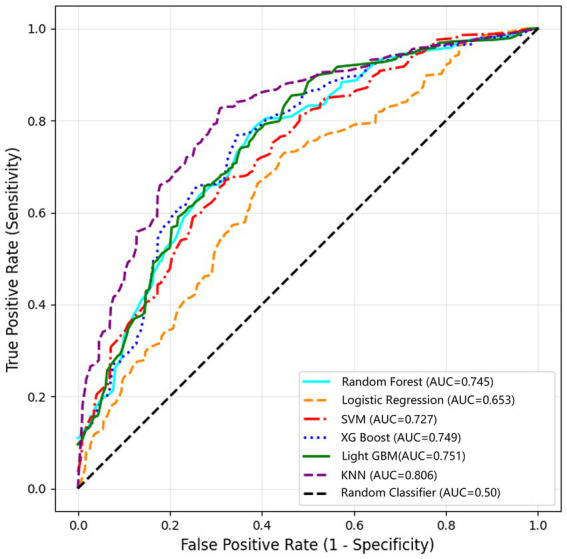
ROC curves of six models trained on Dataset A (1,054 orbital units) using the SMOTE strategy. The dashed diagonal line represents random classifier performance (AUC = 0.50). Higher AUC indicates better discriminative ability. These curves are presented for methodological comparison and to illustrate algorithmic behavior under synthetic oversampling.

**Figure 4:**
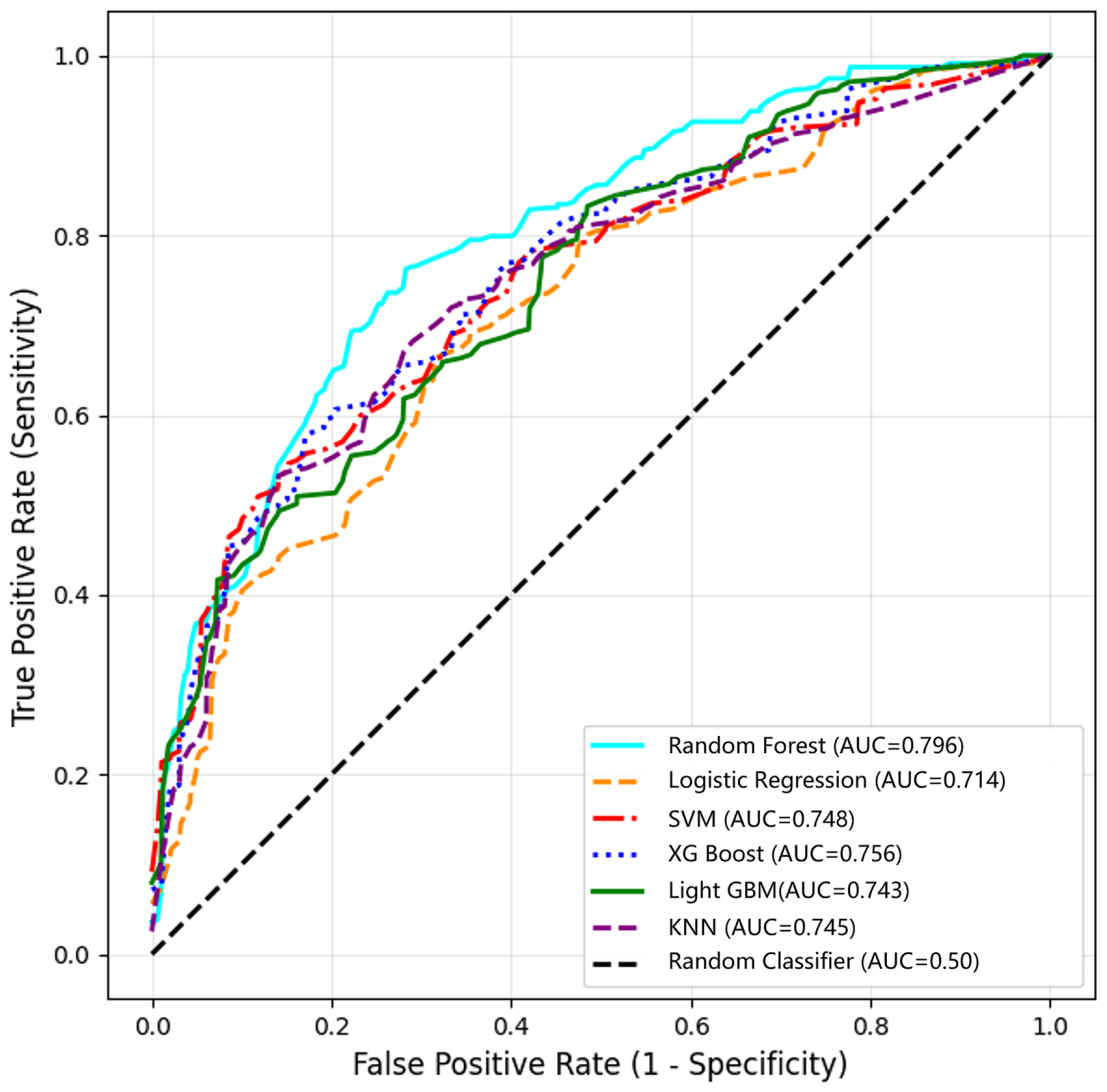
ROC curves of six models trained on Dataset B (886 orbital units) using the SMOTE strategy. The dashed diagonal line represents random classifier performance (AUC = 0.50). Higher AUC indicates better discriminative ability. These curves are presented for methodological comparison and to illustrate algorithmic behavior under synthetic oversampling.

Comparing the two datasets revealed distinct algorithmic sensitivities to data structure. KNN’s nominally high AUC in Dataset A substantially declined in Dataset B, indicating vulnerability to longitudinal redundancy rather than true generalizability. Conversely, Random Forest maintained stable performance, with its F1-score improving when transitioning from the redundant Dataset A to the temporally deduplicated Dataset B. The direct comparison of imbalance strategies on Dataset B confirmed that class weighting provided the highest AUC, whereas SMOTE improved recall, F1-score, and specificity. Given concerns about the physiological plausibility of synthetic samples, class weighting was retained as the preferred strategy for clinical interpretation. Consequently, class weighting is prioritized for clinical severity staging. The ROC curves for all models under the SMOTE strategy are presented in Figures 3, 4 for methodological comparison.

## Discussion

In this study, we developed and evaluated six machine learning models for TED severity stratification using reproducible orbital anatomical features derived from standard MRI. Two data strategies were compared: Dataset A incorporated all available MRI examinations, whereas Dataset B retained only the first MRI examination per patient to reduce temporal redundancy and repeat-visit bias. Our findings indicate that both data structure and algorithm choice critically influence model reliability.

### Impact of data structure on model performance: the role of temporal deduplication and inter-eye correlation

When evaluated under the SMOTE framework, a notable shift in performance rankings was observed between Dataset A and Dataset B, underscoring the sensitivity of algorithmic evaluation to data structure. Dataset A included longitudinal scans from repeated visits, which introduced high intra-patient temporal correlation. In this setting, KNN achieved the highest AUC (0.806) and recall (0.651), likely because serial scans from the same patient are highly similar and may artificially favor proximity-based classification. In Dataset B, which retained only first-visit scans, KNN’s performance declined across all metrics (AUC: 0.745, F1: 0.514), suggesting that its advantage in Dataset A was partly driven by longitudinal redundancy rather than intrinsic feature discriminability (30). Conversely, Random Forest showed improved stability on Dataset B under the SMOTE strategy (AUC: 0.796, recall: 0.669, F1: 0.648). RF’s ensemble architecture, which relies on bootstrap aggregation and random feature subsampling, may be more robust to the removal of temporally correlated samples and better suited to capturing stable anatomical patterns (31, 32).

It is important to note that although Dataset B reduces repeat-visit bias, it still contains bilateral orbital units from the same patients. Because train/test splitting was performed at the orbital level, inter-eye correlation may have introduced minor information leakage. Therefore, the present results should be interpreted as internal validation rather than strict patient-level generalization. Nevertheless, the performance shift between datasets highlights that model estimates can be inflated when intra-patient temporal redundancy is unaccounted for, particularly for distance-based algorithms combined with resampling techniques (26). For clinical deployment targeting patient-level generalization, strict patient-level cross-validation and explicit modeling of inter-eye correlation will be prioritized in future studies. These findings reinforce recent methodological guidelines advocating for transparent reporting of sampling units and patient-aware validation in medical machine learning (33).

#### Anatomical feature-based models: interpretability and clinical relevance

The use of anatomically defined, clinically measurable features enhances the interpretability and translational potential of our models (34). Feature importance rankings were derived from Random Forest models trained under the SMOTE strategy, using Gini importance as the metric. In both datasets, ocular protrusion was identified as the most influential predictor of TED severity, followed by extraocular muscle thicknesses, particularly MRMT and IRMT. This finding is strongly supported by existing literature: proptosis is a core criterion in EUGOGO guidelines for assessing disease activity and severity (8), and MRMT enlargement is the most common imaging manifestation of TED due to its volume and susceptibility to inflammation (35–38). While this prominence reflects proptosis’s explicit role in clinical grading, it does not indicate mere replication of a binary threshold (≥22 mm). Instead, the model learns a non-linear, multi-dimensional integration that dynamically weights protrusion alongside muscle dimensions and supplementary structural parameters. By transforming discrete, threshold-based scoring into continuous probability estimates, the framework mitigates subjectivity in borderline cases and provides a reproducible, quantitative complement to categorical clinical assessment. This design positions the model not as a rule-replication engine, but as a standardized stratification tool that enhances consistency across variable clinical workflows. The consistent prioritization of these established anatomical parameters aligns with current pathophysiological understanding and supports the biological plausibility of our predictions.

Notably, the model also assigned meaningful importance to orbital geometric parameters, including orbital width (w), orbital height (h), and their angular relationships with key anatomical landmarks (w1: w’s angle relative to the optic foramen; h1: h’s angular relationship with the common tendinous ring). While less routinely quantified in clinical practice, these structural dimensions may reflect underlying biomechanical constraints and compartmental remodeling in advanced TED. For instance, orbital width (w) and its angular relationship with the optic foramen (w1) may influence the direction and magnitude of muscle displacement during inflammation. As the medial and inferior recti enlarge, they may exert asymmetric pressure within the conical orbital space, leading to altered nerve traction or compressive optic neuropathy, particularly if the orbital apex is narrow or angularly constrained (39, 40). Similarly, h1, capturing vertical alignment relative to the common tendinous ring, could serve as a proxy for apical crowding—a recognized risk factor for dysthyroid optic neuropathy (41). These hypothesis-generating findings suggest that quantitative orbital geometry may offer supplementary insights into disease progression. Furthermore, LRMT emerged as a significant feature in Dataset B, ranking third. Although traditionally considered less involved, growing evidence links LRMT enlargement to restrictive strabismus and horizontal diplopia (35, 42), suggesting it may represent a stable and generalizable marker of structural involvement across patient cohorts.

Collectively, the consistent importance of all nine features indicates that TED severity is driven by complex, multi-dimensional orbital remodeling rather than isolated changes. Compared to data-driven approaches, such as radiomics or deep learning, relying on high-dimensional, often abstract feature spaces (35, 42, 43), our model offers inherent clinical transparency. By grounding predictions in directly measurable anatomical changes, it enables clinicians to trace the rationale behind model outputs, fostering trust and facilitating seamless integration into routine workflows.

### Comparative performance of machine learning algorithms

A critical methodological consideration emerged in the evaluation of class imbalance strategies. Although SMOTE improved recall and specificity, it does so by generating synthetic samples through interpolation, which may produce anatomically atypical configurations when applied to continuous MRI-derived measurements. In contrast, class-weighted optimization adjusts the decision boundary without altering the original feature distribution. On Dataset B, class-weighted Random Forest achieved the highest AUC (0.811), whereas SMOTE-based Random Forest yielded the highest recall (0.669), F1-score (0.648), and specificity (0.815). We therefore prioritized class weighting as the primary clinical strategy because it preserved the empirical anatomical distribution and achieved the best overall discriminative performance. For clinical severity staging, where minimizing false-positive severe classifications is critical to avoid unnecessary interventions, preserving anatomical integrity and maximizing AUC took precedence over marginal gains in recall obtained through synthetic oversampling. Consequently, class-weighted optimization was designated as the preferred strategy in this study, with SMOTE retained solely for methodological transparency.

The reported performance metrics (AUC: 0.811, F1: 0.648) are conservative relative to recent ensemble or deep learning-based TED classification studies. For instance, Alkhadrawi et al. (44) reported an AUC of 0.929 using automated CT-derived volumetric segmentation combined with an ensemble of Random Forest classifiers and limited patient metadata. While such architecture-level ensemble strategies and hybrid volumetric-metadata pipelines demonstrate strong predictive capacity, our framework emphasizes methodological transparency, data structure rigor, and direct clinical interpretability. First, our evaluation used Dataset B to reduce temporal redundancy from repeat visits. Second, the model relies exclusively on reproducible MRI-derived anatomical measurements rather than automated volumetric pipelines or auxiliary metadata, ensuring that each prediction can be traced to established clinical signs. Third, separating TED into mild, moderate, and severe categories introduces greater morphological overlap than binary or complication-driven endpoints. We therefore acknowledge that architecture-level imbalance handling, such as ensemble voting schemes or class-specific binary classifiers, remains a promising direction for future work, especially if paired with patient-level partitioning and explicit modeling of inter-eye correlation (45). The SMOTE-based ROC curves are presented for methodological comparison and to illustrate algorithmic behavior under synthetic oversampling.

### Limitations and future directions

Several limitations should be acknowledged. First, Dataset B excluded follow-up scans to minimize temporal bias and repeat-visit confounding, thereby sacrificing longitudinal information. While this reduced temporal redundancy, bilateral orbits from the same patients were retained, meaning inter-eye correlations persisted. Our orbital-level partitioning was used to maximize data utilization under the constraints of the available retrospective dataset, but may introduce minor information leakage. Additionally, the intentional inclusion of features aligned with EUGOGO criteria, such as ocular protrusion, ensures clinical interpretability but inherently links model predictions to existing grading standards. While this transparency facilitates adoption, it may limit the model’s ability to identify features independent of current diagnostic thresholds. Future work will explore patient-stratified modeling and mixed-effects approaches to explicitly account for inter-eye correlations and longitudinal clustering. Furthermore, validation against longitudinal treatment response, disease progression, or patient-reported outcomes will be essential to demonstrate prognostic utility beyond established categorical frameworks. Second, although all features were derived from standard MRI planes, manual measurement may introduce inter-rater variability. Future studies could incorporate semi-automated measurement tools to improve reproducibility. Third, this single-center, retrospective study lacks external validation. Multicenter validation is warranted to assess performance across diverse populations and imaging protocols. Finally, while tree-based models performed robustly, emerging architectures such as graph neural networks or hierarchical voting schemes may offer enhanced capability to capture complex patient-level data structures while maintaining clinical interpretability.

## Conclusion

This study demonstrates that data sampling structure and intra-patient correlations significantly influence the evaluation of machine learning models for TED severity prediction. Random Forest with class weighting exhibited the best overall discriminative performance on temporally deduplicated first-visit scans, achieving the highest AUC (0.811) while preserving the original anatomical feature distribution. Ocular protrusion and medial rectus muscle thickness emerged as the primary predictors, reflecting established clinical criteria rather than isolated rule replication. By integrating these parameters with supplementary orbital geometry, the framework transforms discrete clinical thresholds into probabilistic severity stratification, reducing subjectivity in borderline cases. Anchoring predictions to quantifiable measurements and prioritizing methodological transparency provides a standardized, reproducible tool for clinical severity stratification. Future validation will prioritize strict patient-level partitioning and outcome-based endpoints to further assess prognostic utility beyond current classification standards.

## Data Availability

The original contributions presented in the study are included in the article/supplementary material, further inquiries can be directed to the corresponding author/s.
